# An Indirubin Derivative, Indirubin-3′-Monoxime Suppresses Oral Cancer Tumorigenesis through the Downregulation of Survivin

**DOI:** 10.1371/journal.pone.0070198

**Published:** 2013-08-13

**Authors:** Wan-Yu Lo, Nai-Wen Chang

**Affiliations:** 1 Graduate Institute Integrated Medicine, China Medical University, Taichung, Taiwan; 2 Department of Medical Research, China Medical University Hospital, Taichung, Taiwan; 3 Department of Life Sciences, National Chung-Hsing University, Taichung, Taiwan; 4 Department of Biochemistry, College of Medicine, China Medical University, Taichung, Taiwan; Winship Cancer Institute of Emory University, United States of America

## Abstract

Oral cancer is the fourth most common cause of death from cancer in Taiwanese men. Indirubin-3′-monoxime (I3M), a potent cyclin-dependent kinase inhibitor, has therapeutic effects in other cancer cells. In this study, we carried out *in vitro* assays to test cell viability, cell cycle progression, apoptosis, cell migration and invasion in this cancer type. In addition, using an oral tumorigenic animal model, we examined target gene and protein expression using real time qPCR, immunoblotting and immunohistochemical staining. Our results demonstrate that I3M has an anti-proliferative effect in both Cal-27 and HSC-3 oral cancer cell lines and that treatment of Cal-27 and HSC-3 cells with I3M results in apoptosis through the activation of cytochrome *c*. In addition, I3M interrupts the cell cycle in Cal-27 cells in a dose-dependent manner by arresting cells in the G2/M phase. We also found that I3M suppresses migration and invasion in Cal-27 cells by inhibiting the expression of focal adhesion kinase, urokinase-type plasminogen inhibitor, and matrix metalloproteinase 9. Moreover, we identified survivin as a target protein in I3M-treated oral cancer cells. Using an oral cancer mouse model, we demonstrate that topical application of an adhesive gel composed of I3M and poly(vinyl alcohol) (I3M/PVA) has dose-dependent anti-tumorigenic effects. Following treatment, the expression of survivin protein and mRNA was downregulated in cancerous tissues. Furthermore, plasma survivin levels were also reduced in the I3M-treated mice. These results suggest that topical application of I3M, a drug synthesized from indirubin, which is found in Qing-Dai – has therapeutic potential for treating oral cancer.

## Introduction

Oral squamous cell carcinoma (OSCC) accounts for approximately 90% of oral malignancies. Approximately 274,000 new cases are diagnosed annually worldwide, and despite improved diagnostic and therapeutic methods, patients only have a 50% survival rate over 5 years [1]. Smoking, betel-quid chewing, alcohol use, and smokeless tobacco products constitute the major risk factors for oral cancer. Current treatment options for oral cancer include surgery, radiotherapy, and chemotherapy, although the 5-year survival rate for oral cancer remains one of the lowest among common malignant neoplasms [2]. Oral cancer is the sixth most common cancer in Taiwan and the fourth most common cause of death from cancer among Taiwanese men since 2006 [3]. Therefore, the identification of new agents and novel targets for the treatment of oral cancer needed to improve clinical management of this disease.

Danggui Long Hui Wan is a compound from traditional Chinese medicine that is used to treat chronic myelocytic leukemia [4], and the active ingredient appears to be Qing Dai (*Indigo naturalis*), which contains high levels of indigo dye. Furthermore, the anti-leukemic activity of this ingredient has been attributed to the red-colored indigo isomer indirubin. Indirubin and its derivatives strongly inhibit the growth of various human cancer cells, mainly through cell cycle arrest (at G2/M or G1 phase) followed by apoptosis [5], [6]. It has been determined that indirubin derivatives are strong inhibitors of cyclin-dependent kinases (CDKs), glycogen synthase kinase-3β [7], c-Src kinase and STAT3 signaling [8], [9]. Whereas indirubin itself has poor solubility, a low absorption rate, and significant gastrointestinal toxicity, synthetic indirubin-3′-monoxime (I3M) has better pharmacological properties and reduced toxicity. Furthermore, compared with indirubin, I3M inhibits many additional protein kinases as well as STAT3 signaling, and it has been shown to have anti-proliferative effects in vascular smooth muscle cells [10]–[12]. Recently, Indirubin-3′-oxime also have been reported induces mitochondrial dysfunction and triggers growth inhibition and cell cycle arrest in human neuroblastoma cells [13]. Therefore, I3M is considered one of the most potent indirubin derivatives for the treatment of cancer.

Survivin is a critical determinant of cell survival, and it functions both by regulating cell division and inhibiting apoptosis [14]. As a member of the inhibitor of apoptosis (IAP) family of proteins, survivin was originally characterized as a physical caspase inhibitor, providing a cytoprotective step downstream of the death receptor and mitochondrial apoptosis [15]. However, it is now known that X-linked inhibitor of apoptosis protein (XIAP) is the only true physiological inhibitor of caspases 3, 7, and 9 [16]. Despite a lack of structural motifs that mediate caspase binding, survivin can inhibit active caspase 9 through cooperation with the hepatitis B virus X-interacting protein [17]. Moreover, the association of survivin with XIAP leads to a synergistic inhibition of caspase 9 activation [18]. Many studies have shown that survivin is overexpressed in various human cancers and is associated with poor overall prognosis [19]. More specifically, survivin expression is correlated with poor prognosis and chemoresistance in oral cancer [20]–[22]. Furthermore, inhibition of survivin in different head and neck cancers significantly increases the anti-tumorigenic activities of several cytotoxic and targeted therapies [23].

Building upon previous studies, we aimed to study the role of survivin with respect to I3M treatment in oral cancer. In this study, we demonstrate that I3M has multiple anti-tumorigenic activities and that it can inhibit cell proliferation, migration and invasion, while at the same time promoting apoptosis, in oral cancer cells. Using immunoblotting and real-time qPCR analysis, we found that survivin expression was downregulated in the cancer cell line Cal-27 following I3M treatment, identifying survivin as a potential mediator of the anti-tumorigenic activities of I3M. Finally, we verified that I3M inhibits survivin expression and displays anti-tumorigenic activity in an oral tumorigenesis mouse model. Our results suggest that I3M suppresses oral cancer tumorigenesis by mediating the activity of survivin.

## Materials and Methods

### Ethics Statement

The protocol used for the experimental mice was reviewed and approved by the Institutional Animal Care and Use Committee of the China Medical University (IACUC approval no. CMU-99-26-N). All animal studies were conducted according to institutional guidelines (Affidavit of Approval of Animal Use Protocol, No. 98-33-N) approved by the Institutional Animal Care and Use Committee (IACUC) of China Medical University (Taichung, Taiwan).

### Reagents and cell culture

Indirubin, I3M, dimethyl sulfoxide (DMSO), thiazolyl blue tetrazolium bromide (MTT), trypan blue, triton X-100, and penicillin/streptomycin were purchased from Sigma Chemical (St. Louis, MO, USA).

The human oral cancer cell line Cal-27 and the cell line HSC-3 were purchased from the Bioresource Collection and Research Center (BCRC), Food Industry Research and Development Institute (FIRDI) (Hsinchu, Taiwan). Cells were plated in Dulbecco's modified Eagle medium (DMEM; Gibco) and DME/F-12 (Gibco) supplemented with 10% FBS, 100 units/mL penicillin, 100 ng/mL streptomycin, and 1% glutamine at 37°C [24], [25].

### MTT assay

Cell proliferation was assessed using an MTT assay. Cells were seeded into 96-well plates at a concentration of 1,000 cells/well. Six wells were assayed for each experimental treatment. After the cells were treated with 0, 5, or 10 µM indirubin or I3M (dissolved in 0.1% DMSO) for 0, 24, or 48 h, 20 µL of MTT reagent (5 mg/mL; Sigma) was added to each well, and the cells were then incubated for 3 h. The reaction was stop by removing the MTT reagent. DMSO (150 µL) was then added to each well to dissolve the formazan crystals. The absorbance was measured at 570 nm. The effects were also assessed by cell counting using a hemocytometer. All measurements were carried out in triplicate.

### Preparation of subcellular fractions and immunoblotting of cytochrome c

Cells were plated into 10-cm dishes and treated with variable dose I3M for 24 h. After the incubation, the cells were harvested, resuspended in cell extract buffer (20 mM HEPES (pH 7.5), 10 mM KCl, 1.5 mM MgCl_2_, 1 mM EDTA, 1 mM EGTA, and 1 mM dithiothreitol) that containing 250 mM sucrose and protease inhibitor mixture (Roche Molecular Biochemicals, Mannheim, Germany) and homogenized. The homogenates were centrifuged twice at 1,000×*g* for 10 min at 4°C to remove nuclei and unbroken cells. The supernatants were then centrifuged at 10,000×*g* for 15 min at 4°C. The supernatants from the 10,000×*g* spin are referred to as the “cytosolic fraction.” The cytosolic protein samples (30 g) were separated on 12% SDS–PAGE gels and immunolabeled with anti-cytochrome *c* (1∶1000) and anti-β-actin (1∶1000) primary antibodies overnight at 4°C. The second horseradish peroxidase-labeled antibody was incubated with the blots, followed by washing. Chemiluminescent signals were detected using SuperSignal West Femto- Chemiluminescent substrates (Pierce) according to the manufacturer's instructions.

The mitochondrial pellets from the first 10,000×*g* spin were resuspended in cell extract buffer containing 250 mM sucrose to protect mitochondria by 20 strokes using a homogenizer. Homogenates were centrifuged at 750× *g* for 3×10 min at 4°C to remove debris and nuclei. The supernatant was then centrifuged at 15,000× *g* for 20 min; the pellet, which contained “mitochondria fraction”, was lysed in SDS lysis buffer; and 30 µg of mitochondrial proteins was subjected to immunoblot analysis as the above descriptions.

### Annexin V/PI staining

Cal-27 cells (10^5^) were seeded into each well of a 6-well plate and treated with either DMSO or 10 µM I3M for 24 h. The Annexin V-FITC Apoptosis Detection kit (Strong Biotech Corporation, Taiwan) was used to determine the percentage of apoptotic cells. Briefly, the harvested cells were washed with PBS and centrifuged at 200×*g* for 5 min. The cell pellets were resuspended in staining buffer and stained with annexin V-FITC and PI for 15 min at 25°C according to the manufacturer's instructions. The cell samples stained using these reagents could be divided into three populations: apoptotic cells (marked by green fluorescence), dead cells (marked by red or yellow fluorescence resulting from a combination of red and green fluorescence), and live cells (showing little to no fluorescence). The cells were analyzed using a Tali™ Image Cytometer (Invitrogen). The Tali™ Image Cytometer captures 20 images of a stained sample, automatically analyzes the images using digital image-based cell counting and fluorescence-detection algorithms, and displays an accurate quantitative analysis of live, dead, and apoptotic cell populations. All measurements were performed in triplicate.

### Cell cycle analysis

Cal-27 cells were treated with 0, 2.5, 5, or 10 µM I3M, and after 24 h, the cells were washed twice with phosphate-buffered saline (PBS). The cells were fixed overnight with cold 70% ethanol and then stained with a Cycle PI solution consisting of 2 mg/100 mL PBS CAT PI, 1× PBS, 10 mg/mL RNase A, and 5% Triton X-100. Following incubation for 30 min at room temperature in the dark, a FACScan Flow Cytometer was used to detect fluorescence-activated cells. All measurements were made in triplicate.

### Migration determination

Cal-27 cells (10^6^ cells/well) were plated into 6-well plates and incubated for 24 h. The cells were then “wounded” by scratching individual wells using a pipette tip. The cells were incubated with DMEM medium (without FBS) either with or without indirubin and I3M (10 µM). Cells were photographed using phase-contrast microscopy (100× magnification). The migratory ability of the cells was evaluated by measuring the width of the wounds. The migration distances of the cells were derived from the differences between the widths of the wounds at 0, 24, and 48 h.

### Transwell culture system for invasion assays

The invasive abilities of the cancer cells treated with or without I3M were examined using the membrane Transwell culture system. Briefly, we used Transwell membranes (8-µm pore size, 6.5-mm diameter; Corning Costar Corporation) coated with Matrigel for the assays. Cells (1×10^4^ cells) were seeded into the upper wells of the precoated Transwells with I3M (0, 2.5, 5, or 10 µM). The lower Transwells contained the same medium. Following 24 or 48 h incubations, cells from the upper wells and the Matrigel-coated membranes were swabbed with a Q-tip, fixed with methanol, and stained with a 20% Giemsa solution (Sigma). The cells were counted using light microscopy (200× magnification). Three independent experiments were performed in triplicate.

### Immunoblotting

*In vitro studies:* Following each treatment, the cells were isolated to determine the proteins associated with migratory and invasive functions, including P21 (20 kDa) and P53 (53 kDa) (Cell Signaling Technology), survivin (human, 16 kDa), matrix metalloproteinase 9 (MMP-9, 92 kDa) (Thermo Scientific), focal adhesion kinase (FAK, 125 kDa), u-PA(34 KDa), and p-p38 (Santa Cruz Biotechnology). Samples were extracted from the isolated cells (with or without I3M treatment), separated on 12–15% SDS–PAGE gels, and transferred onto PVDF membranes. Chemiluminescent signals were detected as described above for cytochrome *c*. The signals were captured and quantified using the ChemiGenius Bio Imaging System (Syngene).

*In vivo studies:* Plasma samples (40 µg protein) were used to determine the levels of survivin secreted by the tumors using immunoblotting and a survivin (mouse) monoclonal antibody as described above. We used the SwellGel® Blue Albumin Removal Kit (Pierce) to enrich the plasma samples.

### Bioluminescent assays of aaspase-3/7 and -9

A time-dependent study of caspase-3/7 and -9 activities was performed in triplicates using assay kits Caspase-Glo 3/7 and 9 (Promega Corp., Madison, WI, USA) on white 96-well microplate. 10,000 cells per well was seeded and treated with 10 µM of I3M for 12, 24 and 48 hours. Then, caspase activity was investigated according to manufacturer's protocol. Briefly, 100 µL of the caspase-Glo reagent was added and incubated at room temperature for 30 minutes. The presences of active caspases from apoptotic cells will cleave the synthetic tetrapeptide, labeled with aminoluciferin in the reagent. The released aminoluciferin acts as a substrate for the luciferase enzyme, which is measured using Synergy™ 2 Multi-Mode Microplate Reader (Biotek, Winooski, Vermont).

### Preparation of indirubin-3′-oxime/poly(vinyl alcohol) (I3M/PVA) adhesive drug

PVA (10 g; Sigma) was suspended in hot distilled water (100 mL, 90°C) and stirred until completely dissolved. After the polymer appeared to be completely dissolved, the temperature and stirring was maintained for another 4 h to ensure that aggregate were no longer present. The solution was cooled to room temperature, and I3M was added to obtain 10 and 20 µM I3M/PVA adhesive drug mixes.

### Development of the 4-Nitroquinoline 1-oxide (4-NQO) induced oral tumorigenic mouse model

**Figure 1:**
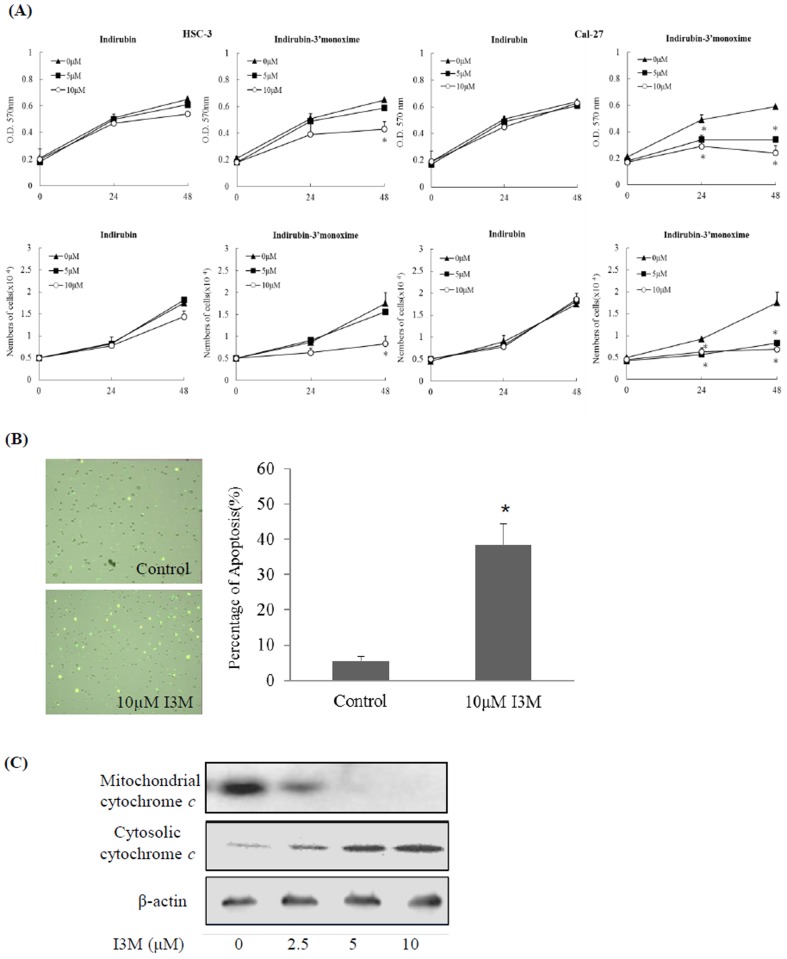
I3M inhibits oral cancer cell proliferation and induces apoptosis. (A) The inhibitory effects of indirubin and I3M on Cal-27 and HSC-3 cell proliferation. Cells were treated for 24 or 48 h with various concentrations of indirubin or I3M. Cell proliferation was analyzed by MTT assay (top) and cell counting using a hemocytometer (bottom). The data are presented as the mean ± S. D. values; asterisks denote a statistically significant difference (*P*<0.05). (B) Cal-27 cells (10^5^) were treated with 10 µM I3M for 24 h, and the percentage of apoptotic cells was determined using the Annexin V-FITC Apoptosis Detection kit. The data are presented as the mean ± S.D. values (n = 3); asterisks denote a statistically significant difference (*P*<0.05) between the I3M treatment and control groups. (C) Immunoblots of protein extracts (30 µg) isolated from the cytosolic and mitochondria fractions of Cal-27 cells treated with 0, 2.5, 5, or 10 µM I3M for 24 h. The results shown are representative of six independent experiments.

**Figure 2:**
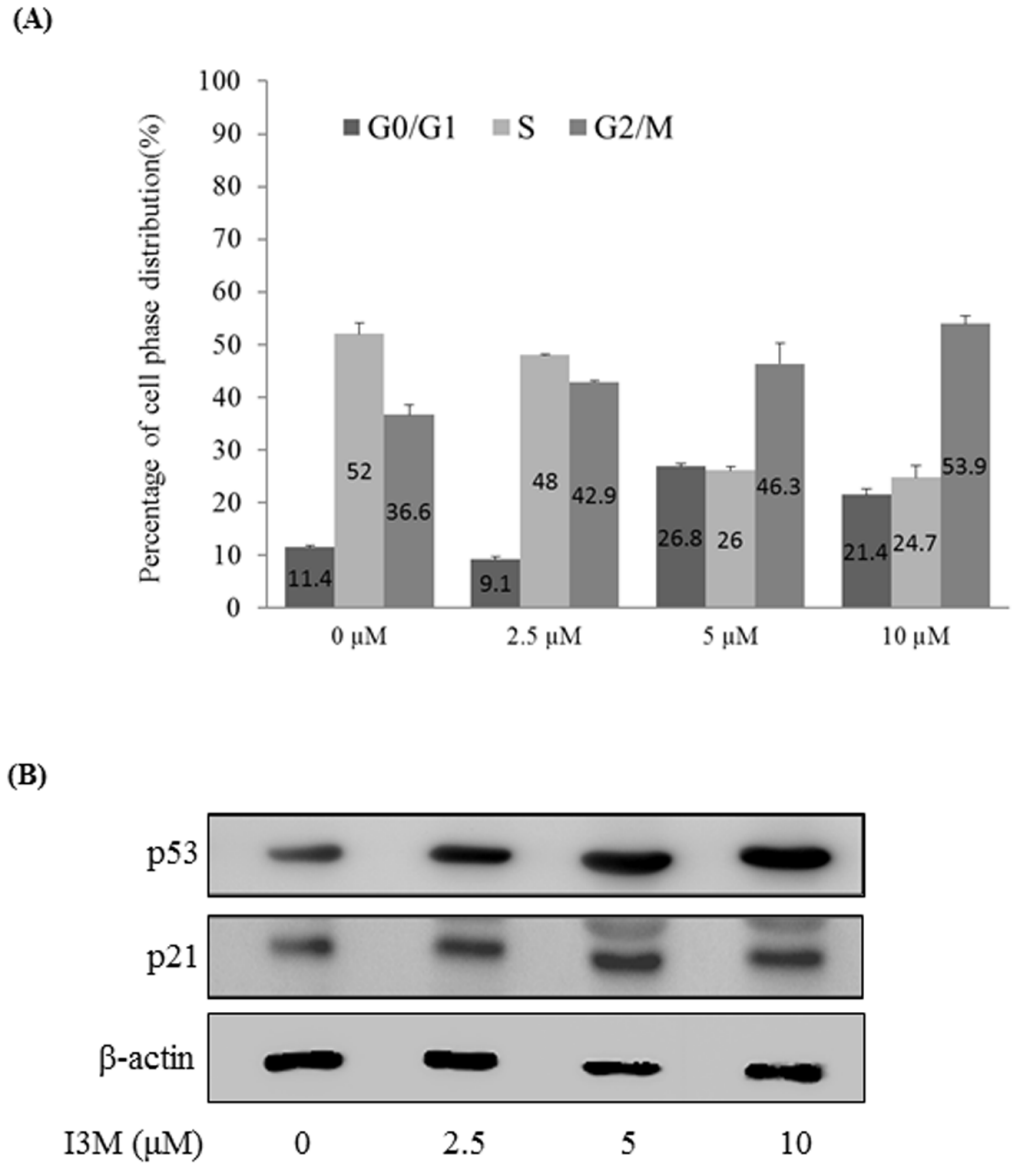
I3M induces cell cycle arrest. (A) Cal-27 cells were treated with I3M (0, 2.5, 5, or 10 µM) for 24 h. Following treatment, the cells were collected, fixed with methanol, stained with propidium iodide, and analyzed using flowcytometry. The data for each sample represent the percentage of cells found in G0/G1, S, and G2/M phases of the cell cycle. (C) Immunoblots showing the expression of cell cycle-related proteins in I3M-treated Cal-27 cells. Total cell lysates were prepared after 24 h treatment with I3M (0, 2.5, 5, or 10 µM). The expression of p53 and p21 was determined using immunoblotting. *β*-actin was used as the loading control in this study. Experiments were repeated three times with similar results.

We evaluated the anti-tumorigenic activity of I3M using an oral tumorigenic model in Six-week-old male C57BL/6JNarl mice (body weight: 21.6±1.2 g). To induce the optimal formation of oral SCCs, we included0.2 mg/mL 4-NQO and 0.5 mg/mL arecoline in the animals' drinking water for 8 weeks as our previously published [26]. Mice (n = 240) were randomized into one of four groups: blank group (n = 60) received only drinking water; Carrier group (n = 60), 10 µM I3M (n = 60) and 20 µM I3M (n = 60) groups received both 4-NQO (200 µg/mL) and arecoline (500 µg/mL) to develop the OSCC animal models. Following the previous study, their drinking water was changed every week, and the mice were allowed access to the water at all times during arecoline/4-NQO treatment, prior to the commencement of the I3M treatments. Following the tumor-induction protocol, which lasted for 8 weeks [24], the tongues and buccal areas of the mice were smeared with PVA alone (carrier group) or with either 10 or 20 µM I3M/PVA every 2 days for 20 weeks (0.1 mg/g mouse body weight), which was initiated after week8 (**Figure S1**, Figure 1 and 2). Topical treatments were initiated at 8 A.M. and were completed within one hour. The treated mice were prohibited from accessing drinking water and food until 12 A.M. All mice were weighed every 4 weeks. The mice (n = 10) were sacrificed every month from each group after week 8; following CO_2_ treatment, an average of 0.9–1.3 mL of heart blood was collected from each mouse, and their tongues were excised whole (with tumors), fixed, embedded, and sectioned for hematoxylin and eosin staining.

### Real-time quantitative polymerase chain reaction (real time qPCR)

Total RNA was extracted from cells using the RNeasy Mini Kit (QIAGEN). The RNA was reverse-transcribed using the SuperScript III First Strand Synthesis System (Invitrogen) according to the manufacturer's instructions. Real-time quantitative PCR was performed using a LightCycler 480 machine and the SYBR Green I Master Kit (Roche Diagnostics) [27]. The primer sequences are listed in Table S1.

### Immunohistochemical analysis

Tissue samples were fixed in 4% paraformaldehyde (Merck) at 4°C. Following a brief wash with PBS, the samples were transferred to 30% sucrose in 0.01 M PBS for cryoprotection. We pre-incubated (2 h, 25°C) 8-µm sections with 10% horse serum and 0.3% Triton X-100 in PBS to prevent non-specific binding. The sections were incubated with specific primary antibodies, including rabbit polyclonal anti-COX2 (1∶200; Abcam), rabbit polyclonal anti-survivin (1∶50, Novus Biologicals), and TUNEL (1∶100; QIA33 Calbiochem, Merck) for 1 h at 37°C followed by overnight incubation at 4°C. The sections were subsequently incubated (2 h, 25°C) with a biotin-conjugated secondary antibody (1∶200; Vector), followed by incubation with an avidin-horseradish peroxidase complex (ABC-Elite).

### Statistical analysis

All experiments were repeated at least three times. All data are presented as the mean ± S.D. values. Student's *t*-test was used to analyze differences between treated and untreated groups. The data were analyzed using the SPSS 12.0 software program. *P*-values<0.05 were considered to be significant.

## Results

### I3M inhibits oral cancer cell proliferation and induces apoptosis

Indigo, indirubin, and I3M were first tested for growth-inhibition activity using the oral cancer cell lines Cal-27 and HSC-3, and the 50% inhibitory concentration (IC_50_) values for these three substances were determined following 24 h drug treatment (Table S2). I3M was clearly more active compared with indigo or indirubin in both cell lines. We then examined the anti-proliferative effects of different concentrations of indirubin and I3M on HSC-3 and Cal-27 cells (Fig. 1A). The MTT assays showed that both 5 and 10 µM indirubin had no obvious anti-proliferative effects on either cell lines after 48 h of treatment. In contrast, 10 µM I3M elicited significant anti-proliferative effects on HSC-3 cells after 48 h, and both 5 and 10 µM I3M exerted anti-proliferative effects on Cal-27 cells. The early stages of apoptosis were quantified using FITC-conjugated annexin V and analyzed with a Tali™ Image Cytometer. In Cal-27 cells, 38.5% of cells were positive for annexin V following 24 hours of treatment with I3M (10 µM) compared with only 5% of cells in the control group (Fig. 1B). After 24 h I3M treatment, the dose-dependent increasing of immunoblotting was evident in the cytosolic fractions of cytochrome *c* (Fig. 1B).

### I3M induces cell cycle arrest largely at the G2/M phase and increases p53 and p21^WAF1^ expression

To determine whether I3M induces Cal-27 cell cycle arrest, we used flow cytometry to analyze how increasing concentrations of I3M affect progression of the cell cycle. Treatment with I3M over 24 h increased the proportion of Cal-27 cells remaining in G2/M phase in a dose-dependent manner. Specifically, the percentage of G2/M-phase cells increased from 36.6% to 53.9% in response to 10 µM I3M. In addition, we noted an increase in the percentage of G0/G1-phase cells from 11.4% to 21.4% (Fig. 2A). It is known that p53 suppresses tumor growth via cell cycle arrest or triggering apoptosis. Therefore, we used immunoblotting to determine whether I3M treatment modulates the expression levels of p53. Following 24-h treatment with different concentrations of I3M, Cal-27 cells exhibited dose-dependent increases in p53 expression, with concurrent increases in p21^WAF1^ protein expression as well, suggesting that one of the mechanisms by which I3M exerts its anti-proliferative effects could be mediated by p53 (Fig. 2B).

### I3M inhibits cancer cell migration and invasion

**Figure 3:**
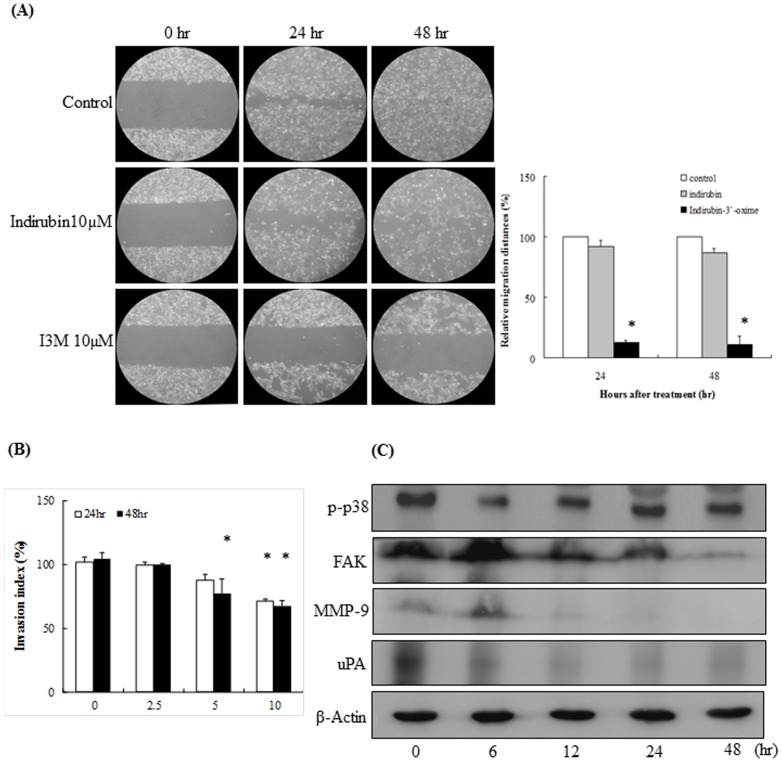
I3M inhibits cancer cell migration and invasion. (A) Incubation of cells in the absence or presence of 10 µM indirubin or I3M was carried out for 0, 24, or 48 h. The cells were photographed under phase-contrast microscopy (100× magnification). The data are shown as the percentage of inhibition; asterisks indicate a statistically significant difference (*P*<0.05) between treatment and control groups. (B) Cells (10^4^) were plated in the upper chamber with I3M (0, 2.5, 5, or 10 µM) and allowed to undergo migration for 24 or 48 h. Quantification of cells in the lower chamber was carried out by counting cells under ×200 magnification. The percentage of inhibition and column mean values were derived from 3 independent experiments. The asterisks indicate a statistically significant difference (*P*<0.05) between treatment and control groups. (C) Immunoblots showing changes in the levels pp38, FAK, MMP-9 and uPA proteins associated with migration and invasion in Cal-27 cells following 10-µM I3M treatment (n = 3).

To examine whether I3M inhibits Cal-27 cell invasion, we performed a wound healing assay. As shown in Fig. 3A, migration distances were significantly decreased in cells treated with 10 µM I3M compared with the control and indirubin groups. Next, we carried out a Matrigel Transwell Assay to determine whether I3M also affects cell invasion. We found that a 10-µM dose of I3M significantly inhibited cell invasion at both 24 and 48 h. We also observed similar results at a lower I3M dosage (5 µM) following 48 h of treatment (Fig. 3B). To identify the specific molecules affected by I3M, we performed immunoblotting analysis to determine the expression levels of several proteins involved in invasion and migration. The levels of FAK, MMP-9, and urokinase-type plasminogen activator (uPA) were significantly reduced after I3M treatment for 6 h. However, I3M induced a persistent activation of phosphor-p38 (Fig. 3C). These results suggest that changes in the expression levels of FAK, MMP-9, and uPA play a role in the inhibition of invasion and migration observed in I3M-treated Cal-27 cells.

### Identification of survivin as a target of I3M

**Figure 4:**
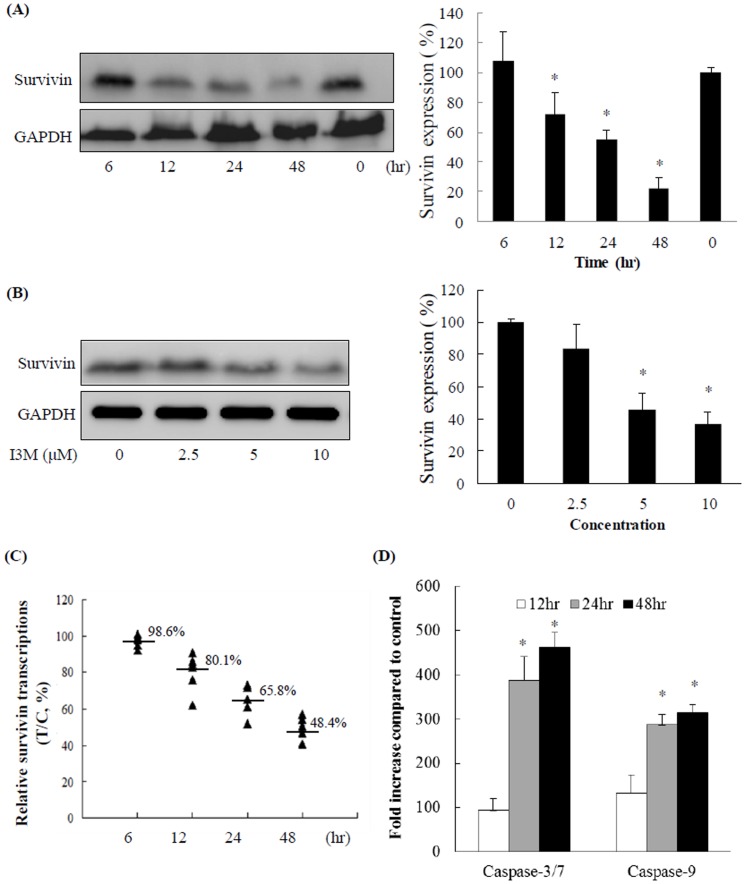
Identification of survivin is a target of I3M treatment and associate with Caspase-3/7 and -9 activity. (A) A demonstration of time-dependent survivin downregulation following 10-µM I3M treatment for various durations. Data are presented as the mean ± S. D. values (n = 3); asterisks indicate a statistically significant difference (*P*<0.05) between the treatment and control groups. (B) The dose-dependent of downregulation of survivin following I3M treatment for 12 h. The data are presented as the mean ± S. D. values (n = 3); asterisks indicate a statistically significant difference (*P*<0.05) between treatment and control groups. (C) Cal-27 cells were incubated with I3M (10 µM) for 0, 6, 12, 24, or 48 h. Shown are the relative gene expression levels for each sample in the treatment group (n = 6). The bold lines denote the mean related percentage of the individual treatment groups. T/C: Treated group (6, 12, 24, or 48 h)/control group (0 h). (D) A demonstration of time-dependent Caspase-3/7 and -9 upregulations following 10-µM I3M treatment for 12, 24, and 48 h.

An immunoblotting analysis demonstrated both time- and dose-dependent downregulation of survivin by I3M (Fig. 4A and 4B). Survivin expression was significantly reduced following treatment with 10 µM I3M from 12 to 48 h. Real-time qPCR also showed downregulation of survivin mRNA levels in I3M-treated cells, suggesting that I3M suppresses survivin expression at the transcriptional level (Fig. 4C). To examine the downregulation of survivin by I3M have any effect on caspase-3/7 and -9 activities. We measured the caspase-3/7, and -9 activities of 10 µM I3M treatment at 12, 24 and 48 h time-point. The result show the very significant increased in caspase-3/7 and -9 activities were detected after 12 (94 fold and 131 fold), 24 (388 fold and 282 fold) and 48 h (462 fold and 314 fold) of I3M exposure. Thus, these data suggest that I3M not only suppresses survivin in Cal-27 cells, also affects the intrinsic (mitochondrial-caspase-9) pathway.

### I3M suppresses 4-NQO/arecoline-induced oral cancer in mice

**Figure 5:**
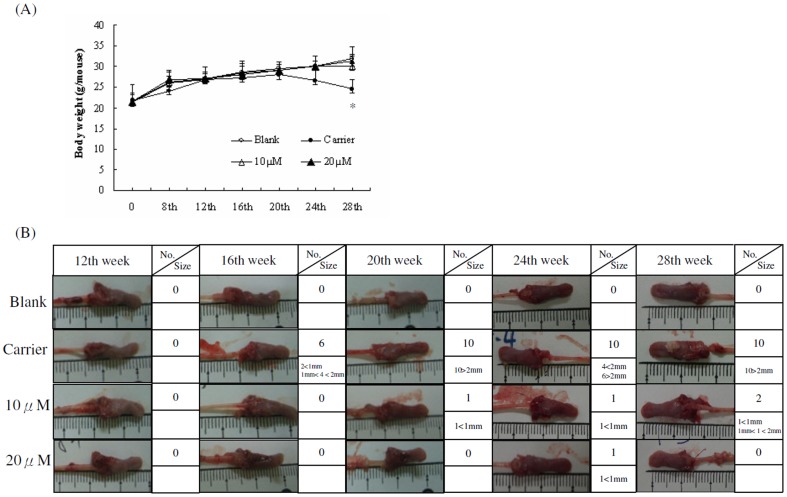
I3M suppresses 4-NQO/arecoline-induced oral cancer in mice. (A) In the mice experiments, the body weight were not shown the significant different among the four groups until 8 th week. After the I3M experiments, the body weight of the carrier group was significantly lower than any other groups at 28 th week (*p* = 0.031, n = 10). (B) Oral cancer lesions were induced on the tongues of the mice with 0.5 mg/mL arecoline and 0.2 mg/mL 4-NQO. The table shows tumor numbers and sizes from four groups (10 mice/group/sampling).

Water consumptions were reported weekly in each group over the 28-week, the time course (4weeks/time point) were shown in Figure S1. The consumptions were observed among the experimental groups. Mice offered water with 4-NQO (200 µg/mL) and arecoline(500 µg/mL) consumed the less amount of water before week 8 (carrier, 10 µM, and 20M groups) than the blank group. However, the least amount of water at 20, 24 and 28 week is the carrier group. At 8 weeks, the body weight was lower in the carrier group mice (Fig. 5A). When 4-NQO and arecoline administrations were discontinued and mice were given tap water at week 9, both water intake and body weight of mice increased. By week 12, 16, 20 and 24, there were no significant differences in body weight among the four groups. At week 28, the body weight was significant lower in carrier group than in any other group. Mice treated with carrier alone exhibited a time-dependent increase in both the number and size of their tongue tumors. Strikingly, local treatment consisting of 10 or 20 µM I3M/PVA gel smeared on the tongues of the mice suppressed tumorigenesis, with higher concentrations showing greater effectiveness (Fig. 5B).

### I3M decreases survivin and COX-2 expression while increasing the percentage of TUNEL-positive cells *in vivo*

**Figure 6:**
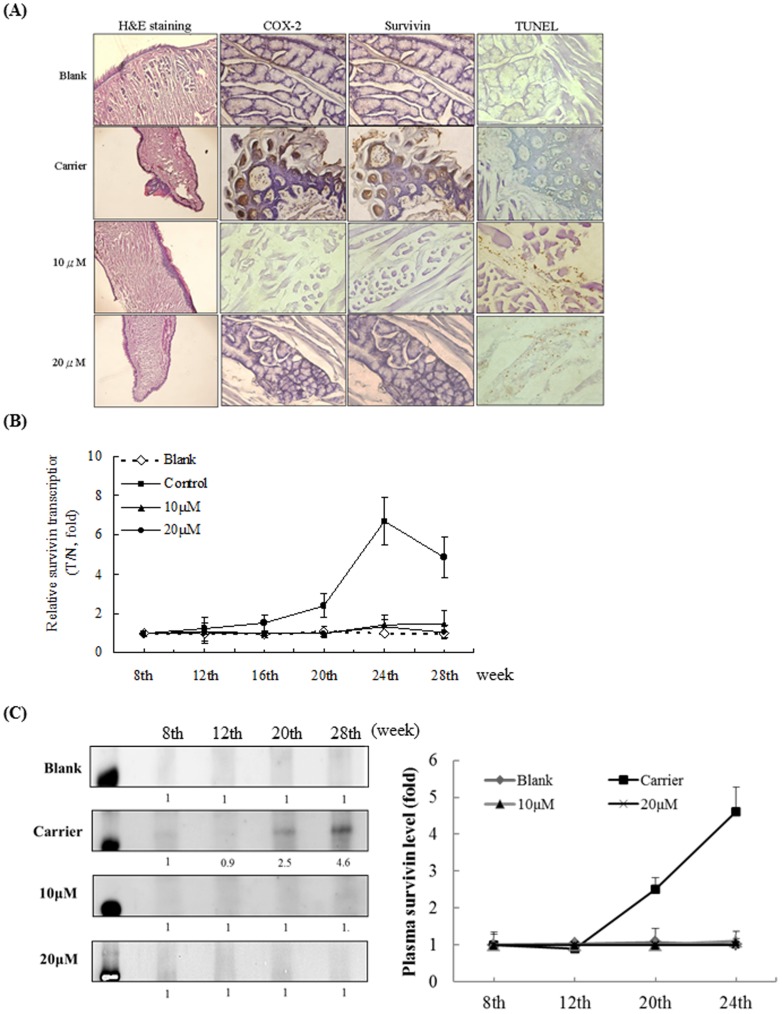
I3M downregulates expression of survivin and COX-2 and increases the number of TUNEL-positive cells *in vivo*. (A) Immunohistochemical staining of COX-2 and survivin expression at 28 weeks. (B) Relative gene expression levels of *survivin*, as determined by real-time quantitative PCR. Carrier-treated mice exhibited an increase in *survivin* mRNA expression from week 20 onwards. T/C: Treated group/Control group (n = 10). (C) Plasma was collected from mice, and the plasma survivin levels were determined by Immunoblotting. The densitometry data presented below the bands are fold-change values relative to a tumor-progressive control (week 4) (n = 10).

H&E staining at different time points showed that 4-NOQ/arecoline exposure increased SCC formation in carrier-treated mice. In contrast, I3M decreased SCC formation after 28 weeks in a dose-dependent manner. Immunohistochemical staining showed high levels of both COX-2 and survivin in SCCs in carrier-treated mice at 28 weeks. Notably, I3M caused dose-dependent decreases in survivin and COX-2 expression. A TUNEL assay was carried out to assess the levels of apoptosis occurring *in vivo*. As anticipated, the proportion of TUNEL-positive cells was markedly increased in the 10- and 20-µM I3M treatment groups compared with the carrier group (Fig. 6A). Relative expression of survivin mRNA levels began to increase at 16 weeks in the carrier group, consistent with the initiation of tumor formation at that time. In contrast, both the 10 and 20 µM I3M treatments suppressed survivin mRNA expression (Fig. 6B). To test the effect of I3M treatment on plasma survivin protein levels, a Western blotting analysis was performed at different time points (Fig. 6C). We determined that survivin levels had increased 2.5-fold by 20 weeks and 4.6-fold by 28 weeks in the carrier group, consistent with the mRNA expression levels shown in Figure 6B. In contrast, in both the 10- and 20-µM I3M treatment groups, the plasma levels of survivin protein had decreased significantly.

## Discussion

I3M has been shown to have a broad spectrum of anti-tumorigenic activities in many human cancer cells [12]. In this study, we confirmed that I3M possesses a number of anti-tumorigenic activities *in vitro*, and we identified a novel anti-tumorigenic role for I3M *in vivo*. Importantly, our data suggest that I3M inhibits survivin expression in oral cancer.

Initially identified as a potent CDK inhibitor that interacts with the ATP-binding site of the kinase, I3M causes cell cycle arrest at G2/M phase (at higher I3M concentrations) or at G1 phase (at lower I3M concentrations) in several cancer cell lines, events which are often followed by apoptosis [5], [6]. Our data show that 10 µM I3M treatment predominantly caused the arrest of Cal-27 cells at the G2/M phase. Shi et al. have shown evidence that I3M mainly elicits apoptosis through an extrinsic pathway with a type II response mediated by the pro-apoptotic Bid and Bax proteins [28]. In their study, it was shown that I3M induced both p53 and p21^WAF1^ expression in HeLa cells, which is consistent with our findings.

Migration and invasion are key aspects of cancer metastasis. The misregulation of several protease enzymes is responsible for invasive cell migration [29]. One of the key proteases, uPA, can cleave the extracellular matrix and mediate the conversion of plasminogen to plasmin. In turn, plasmin mediates invasion directly by degrading matrix proteins and activating MMP [30]. In addition, uPA exerts non-proteolytic effects by interacting with the uPA receptor (uPAR) to regulate cellular/extracellular interactions as an adhesion molecule for vitronectin [31]. The expression of uPA/uPAR is correlated with invasive cancer phenotypes and poor prognosis [32]. Our data show that I3M also inhibits the expression of uPA and MMP-9, which is well correlated with the ability of I3M to inhibit migration and invasion in Cal-27 cells. FAK is a non-receptor tyrosine kinase important for tumor initiation and progression. Epithelial cells are capable of transforming in the absence of FAK, but they do not undergo malignant conversion to invasive carcinomas [33]. In this study, we show that FAK expression was abolished in the presence of I3M, which is consistent with the ability of I3M to inhibit tumorigenesis. Recently, Kim et al. [34] also reported inhibition of MMP-9 expression and FAK activation by an indirubin derivative in head and neck cancer cells. We found that I3M activates p38 mitogen-activated protein kinase (MAPK), consistent with the findings of Zhen et al. [35]. Although p38 MAPK activation is required for the expression of uPA/uPAR during breast cancer [36], the ability of I3M to stimulate persistent p38 phosphorylation appears to be distinct from the downregulation of uPA in Cal-27 cells. Recently, Mehrotra S et al. reported that the survivin-XIAP complex could activate NFκB, leading to increased fibronectin gene expression via β1 integrin signaling and the activation of FAK and Src [37]; furthermore, they concluded that IAPs are direct metastasis genes that act independently of IAP inhibition of cell death. Our study notes that I3M can inhibit survivin expression in oral cancer cells, a finding that could explain the ability of I3M to inhibit Cal-27 migration and invasion.

The *in vivo* OSCC tumorigenesis model used in this study utilized two carcinogens: 4-NQO and arecoline. Several reports have suggested that 4-NQO treatment can induce all the stages of oral carcinogenesis and that it causes similar histological and molecular changes in mouse and human oral cancers [38]. The other carcinogen, arecoline, is one of the major alkaloids found in the areca nut. Co-administration of both carcinogens was intended to mimic certain human behaviors and was 100% effective at inducing tongue tumors in mice [26]. Interestingly, in the rat, 4-NQO can induce survivin expression in certain tongue tumor cells [39], which we also observed in our 4-NQO/arecoline-treated mice. Our data suggest that survivin expression and oral tumorigenesis be inhibited by topical application of I3M. Using an *in vivo* apoptotic assay, we show increased numbers of TUNEL-positive cells in I3M-treated tumors, an exciting finding that reflects the potential for use of I3M as a survivin inhibitor. In addition, we noted that COX-2 induction was sometimes observed in the tumors of our *in vivo* model and that I3M was able to inhibit this induction. Because COX-2 is involved in tumorigenesis and its inhibition suppresses oral cancer invasiveness [40], [41], the mechanisms by which I3M inhibit tumorigenesis are manifold.

Oral cancer is frequently a locally advanced disease with very high recurrence and mortality rates [2]. Attempts to use survivin-based vaccine therapies to treat advanced or recurrent oral cancers have not been successful [42]. However, using local treatment, we were able to apply very high concentrations of I3M to the tumor sites in order to take advantage of its anti-survivin and anti-tumorigenic activities while avoiding systemic toxicity. This type of oral cancer therapy is particularly appealing in cases where anatomic limitations or poor patient performance make surgery impossible. In addition, oral dysplasia, also known as a pre-cancerous lesion, may benefit in particular from topical treatment. One previous study has suggested that survivin, MMP-9, loss of heterozygosity, and altered DNA content are all potential markers for the progression from dysplasia to cancer [43]. In our study, the expression of both MMP-9 and survivin were downregulated by I3M and tumorigenesis was potently suppressed by higher doses of I3M (20 µM). Therefore, it may be more effective to treat patients at the dysplasia stage. Our encouraging *in vivo* results provide strong evidence for the design of a clinical trial that would administer local therapy to suitable patients. Similarly, local application of the anti-survivin agent terameprocol (EM-1421) has been used to treat HPV-linked cervical intraepithelial neoplasia [44]. Plasma survivin levels are a prognostic biomarker for several cancers. Higher plasma survivin levels are associated with increased metastasis in ovarian cancer, increased nodal involvements in breast cancer, and increased aggressive behaviors in prostate cancer [45]–[47].

An important implication from this study is that plasma survivin levels can be used as a biomarker to evaluate the response to I3M treatment, as I3M clearly downregulated plasma surviving in our model. Taken together, the results of our study provide both *in vitro* and *in vivo* evidence that I3M has the potential to become a multi-target drug in anticancer therapy due to its ability to target diverse mechanisms involved in proliferation, the cell cycle, apoptosis, migration, invasion, and tumorigenesis. Furthermore, this study suggests that topical I3M treatment may be a useful tool in future clinical trials aimed at oral cancer.

## Supporting Information

Figure S1 **Development of the 4-Nitroquinoline 1-oxide (4-NQO) induced oral tumorigenic mouse model.** (A) The flowchart of animal model. (B) Smear over the tongue every 2 day. (C) The water intakes of mice were reported.
(DOCX)

Table S1 **Quantitative PCR (qPCR) primers and probes used for specific gene quantification.**
(DOCX)

Table S2 **Growth inhibition of Indigo, indirubin and Indirubin-3′-monoxime for 24 hr on human OSCC cells.**
(DOCX)
