# Supplementary figures and images for: An Indirubin Derivative, Indirubin-3′-Monoxime Suppresses Oral Cancer Tumorigenesis through the Downregulation of Survivin
Source: PLoS One. 2013 Aug 13;8(8):e70198. doi: 10.1371/journal.pone.0070198 (PMC3742732; doi:10.1371/journal.pone.0070198)

**Figure S1**

**(A)**


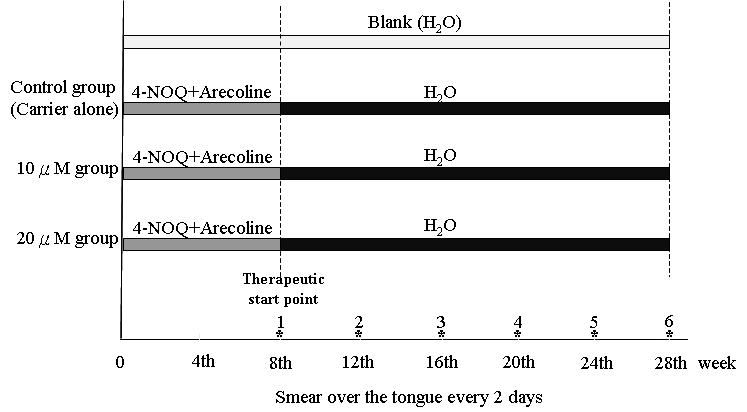


**(B)**


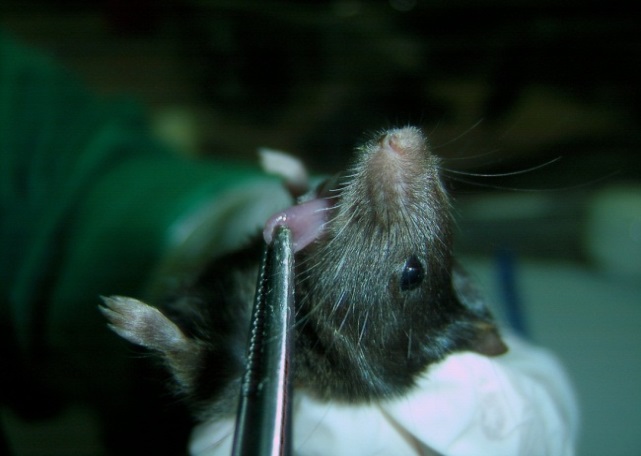

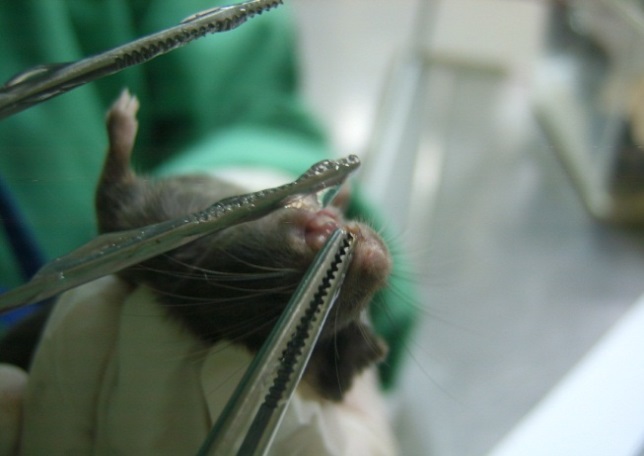


**(C)**


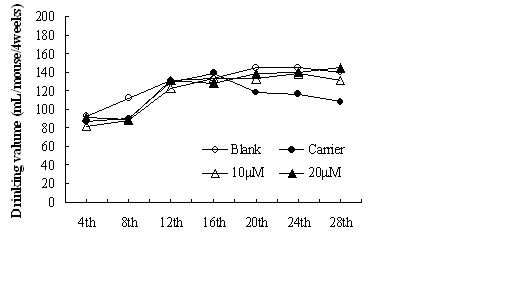


week

Supplement: Figure S1 — Development of the 4-Nitroquinoline 1-oxide (4-NQO) induced oral tumorigenic mouse model. (A) The flowchart of animal model. (B) Smear over the tongue every 2 day. (C) The water intakes of mice were reported. (DOCX) [file pone.0070198.s001.docx]
