# Supplementary material for: An Indirubin Derivative, Indirubin-3′-Monoxime Suppresses Oral Cancer Tumorigenesis through the Downregulation of Survivin
Source: PLoS One. 2013 Aug 13;8(8):e70198. doi: 10.1371/journal.pone.0070198 (PMC3742732; doi:10.1371/journal.pone.0070198)
Supplement: Table S1 — Quantitative PCR (qPCR) primers and probes used for specific gene quantification. (DOCX) [file pone.0070198.s002.docx]

**Table S1**

| **Protein acces No.** | **Gene acces**  **No.** | **Gene name** | **Primer**  **name** | **Sequence**  **(5’→3’)** | **UPL**  **probe no.** |
| --- | --- | --- | --- | --- | --- |
| O15392 (Human) | [NM_001012270](https://qpcr.probefinder.com/showsequence.jsp;jsessionid=96391BDB78CB911C6E29B6E9110D3AAD?seqNo=305523) | *BIRC5* | BIRC5-F | gcccagtgtttcttctgctt | #11 |
|  |  |  | BIRC5-R | ccggacgaatgctttttatg |  |
| P04406  (Human) | NM_002046.3 | *GAPDH* | GAPDH-F | ctctgctcctcctgttcgac | #60 |
|  |  |  | GAPDH-R | acgaccaaatccgttgactc |  |
| O70201  (mouse) | [NM_009689.2](https://qpcr.probefinder.com/showsequence.jsp;jsessionid=AF37C93C02852A2666904BDA1BE530A4?seqNo=774664918) | *Birc5* | mBirc5-F | cccgatgacaacccgata | #71 |
|  |  |  | mBirc5-R | catctgcttcttgacagtgagg |  |
| P16858  (mouse) | NM_008084.2 | *GAPDH* | mGAPDH-F | caatgaatacggctacagcaac | #77 |
|  |  |  | mGAPDH-R | ttactccttggaggccatgt |  |

**UPL probe: Roche universal probe for LightCycler 480 system**
