# Supplementary material for: An Indirubin Derivative, Indirubin-3′-Monoxime Suppresses Oral Cancer Tumorigenesis through the Downregulation of Survivin
Source: PLoS One. 2013 Aug 13;8(8):e70198. doi: 10.1371/journal.pone.0070198 (PMC3742732; doi:10.1371/journal.pone.0070198)
Supplement: Table S2 — Growth inhibition of Indigo, indirubin and Indirubin-3′-monoxime for 24 hr on human OSCC cells. (DOCX) [file pone.0070198.s003.docx]

**Table S2**

| **Compounds** | **Growth inhibition, IC_50_ (μM)^a^** | |
| --- | --- | --- |
|  | 24h treatment | |
|  | CAL-27 | HSC-3 |
| Indigo | 88.5±12.3 | 92.6±9.2 |
| Indirubin | 42.2±6.7 | 50±9.5 |
| Indirubin-3′-monoxime | 4.8±0.9 | 12.5±0.8 |

^a^ Growth inhibition was determined using the Sulforhodamine B assay. IC_50_ values were calculated as survival of treated cells over control cells x100 (T/C %). Values are given as mean± S.D. of six separate assay.
